# Is collagenase-3 (MMP-13) expression in chondrosarcoma of the jaws a true marker for tumor aggressiveness?

**DOI:** 10.1186/1746-1596-3-26

**Published:** 2008-06-13

**Authors:** Manal M Zyada, Ali A Shamaa

**Affiliations:** 1 Oral Pathology Department, Faculty of Dentistry, Mansoura University, Mansoura, Egypt; 2 Oral Biology Department, Faculty of Dentistry, Minia University, Minia, Egypt

## Abstract

**Background:**

Matrix metalloproteinases (MMPs) play an important role in the modeling and remodeling of the extracellular matrix in both physiologic and pathologic states and thus plays an important role in tumor progression. Human collagenase-3 (MMP-13) is a member of matrix metalloproteinase family of enzymes that was originally identified in breast carcinomas and subsequently detected during fetal ossification and in arthritic processes.

**Aim:**

The present study was designed to investigate the expression MMP-13 and to correlate its expression with clinicopathological parameters in chondrosarcoma of the jaws.

**Methods:**

Archival tumor tissues from 11 patients with chondrosarcoma of the jaws were analyzed by immunohistochemistry for the expression of MMP-13. Clinical information was obtained through the computerized retrospective database from the tumor registry between 1998 to 2006.

**Results:**

Eight of 11 cases (72.8 %) of chondrosarcomas showed a positive reaction for MMP-13, whereas two cases of normal cartilage were negative for this collagenase. As regard the clinicopathological parameters, there was no correlation between MMP-13 expression and sex, age and tumor site. While, there were significant associations between MMP-13 expression and both of mitotic counts and necrosis. On the other hand, there was a significant difference between low and high grade tumors (P < 0.05) regarding MMP-13 expression. Also, there was no significant correlation between MMP-13 expression in primary lesions and their local recurrence.

**Conclusion:**

MMP-13 is expressed in the majority of chondrosarcoma of the jaws. It is also noteworthy that the expression of MMP-13 may be related to tumor biological aggressiveness and used to aid in predicting patient's poor prognosis.

## Background

Chondrosarcoma, a malignant cartilage-forming mesenchymal tumor, displays a wide range of clinical behavior that can be difficult to predict with histological analysis [1,2]. These tumors account for approximately 25% of all malignant tumors arising from the skeletal system [3], but are considered by most authorities to involve the jaws only rarely; probably less than 1% of all chondrosarcomas arise in the head and neck area [4].

Controversies surrounding the relationship between chondrosarcoma of the jaws and chondrosarcoma of the skeleton have revolved around their biologic behavior, histopathological features and clinical response to conservative therapy. Chondrosarcoma of jaws is locally aggressive with a high recurrence rate but rarely metastasize to distant sites [5]. If chondrosarcoma spreads from its primary site (i.e., metastasizes), it usually spreads to the lungs. Metastasis is rare with low-grade tumors, but has been seen, even up to 10 years after diagnosis [6]. About half of grade III and nearly all dedifferentiated chondrosarcomas will metastasize. Up to 13% of recurrent chondrosarcomas are of a higher grade than the original neoplasm [7,8].

Conventional chondrosarcomas can be categorized according to their location in bone into central, peripheral, and juxtacortical chondrosarcomas. Rare subtypes of chondrosarcoma, including dedifferentiated, mesenchymal, and clear cell chondrosarcoma are discerned, together constituting 10%–15% of all chondrosarcomas [9]. Clear cell chondrosarcoma is a low -grade malignant tumor and tends to metastasis rarely [10]. While, aggressive chondrosarcoma subtypes, such as dedifferentiated and mesenchymal chondrosarcomas bear a poor prognosis [11].

Histological grading is the best and most commonly used marker at present for the prognosis of chondrosarcomas; however, it is also subject to interobserver variability [12]. This is worrisome, because therapy for grade I and grade II chondrosarcoma may differ. Wide surgical excision remains the best available treatment for intermediate-to high-grade tumors [9]. Most treatment failures are due to local recurrences [5].

Currently, treatment decisions in chondrosarcomas are guided by clinical-pathological factors such as age, sex, metastasis, recurrence, and histological grade. Although useful, these factors fail to provide definitive information regarding the overall aggressiveness of a tumor and its potential to recur. In theory, a factor that consistently identified patients at risk for recurrent disease would help to improve disease-free survival by allowing physicians to select high-risk patients for more aggressive treatment. In addition, identification of a biological marker of aggressive disease would help to provide new avenues for rational drug design targeted against specific molecular defects [13].

In recent years, several biologic markers have been identified that they may provide additional diagnostic and prognostic information in the management of chondrosarcomas [14].

The matrix metalloproteinases (MMPs) comprise a family of at least 25 enzymes that degrade the extracellular matrix [15]. These enzymes operate during normal development in tissue differentiation and remodeling [16,17], but are also active under pathological conditions to cause inflammatory disease, degradation of bone [18] and cartilage [19], as well as tumor metastasis [20,21]. They are secreted as inactive latent forms, depend upon a zinc-binding active site, and are inhibited by specific proteins referred to as the tissue inhibitors of matrix metalloproteinases or TIMPs [22-24]. In general, these MMPs enzymes are classified according to their substrate specificity and structural similarities [25-27]. There are four major subgroups: (a) the collagenases degrade collagen types I, II and III [25], (b) the gelatinases act on types IV, V, VII and X collagens [28], (c) elastin and the stromelysins shows a broader specificity, acting on proteoglycans, laminin, and fibronection [26] and (d) MT-MMPs that represent membrane-bound forms of the enzymes, which activate latent MMP-2 and can cleave collagen at the classic site [29]. MMPs expression is differentially regulated by several growth factors [18].

Human collagenase-3 (MMP-13) is a recently identified member of the matrix metalloproteinase (MMP) family that was originally isolated from breast carcinoma [30]. Biochemical characterization of colloagenase-3 has revealed that it is a very potent enzyme that, after activation through a proteolytic cascade mechanism, displays a broad spectrum of activity against connective tissue components [31]. In addition to its proteolytic activity on fibrillar collagens, collagenase-3 is also a powerful gelatinase and may thus contribute to further degradation the initial cleavage products of collagenolysis to small fragments suitable for further metabolism [32]. Very recent studies have provided that collagenase-3 may also degrade the large cartilage proteoglycan aggrecan [33] and other components of the extracellular matrix and basement membranes, including type IV collagen, fibronectin, and tenascin [34].

Preliminary studies on the prognostic value of collagenase-3 in breast cancer have revealed that this enzyme is a marker of poor clinical outcome in breast cancer patients [35]. In addition, recent studies have shown that collagenase-3 is also over-expressed by a subset of squamous cell carcinomas with extensive local invasion [36-38], but has only rarely been described in connective tissue tumors [39,40]. To date, there have been no reported among the English language literature describing the possible production of this enzyme by chondrosarcoma of the jaws. In this regard, the finding that collagenase-3 is produced by chondrocytes during human fetal development [41,42] and in joint-destructive processes [43,44] prompted us to examine the possibility that this enzyme could be also associated with tumor processes involving these cells. The present study was designed to evaluate MMP-13 immunohistochemistry for its presence in a series of chondrosarcoma of the jaws as well as normal cartilage. It is also aimed to investigate relation between its expression and clinico-pathological parameters of this tumor.

## Methods

Patients with chondrosarcoma of the jaws were treated by surgical resection as a primary means of disease control. Of the treated patients, eleven had archival paraffin-embedded specimens and adequate clinical history to enable the following study. They were selected from paraffin blocks archives of Oral and General Pathology Departments, Faculty of Dentistry and Faculty of Medicine, Mansoura University between 1998 to 2006. All cases did not previously receive chemo-or radiotherapy before the operation. Chondrosarcomas were graded according to WHO classification [45]. The grading is based primarily on nuclear size of tumor cells, nuclear staining (hyperchromasia, or darker staining of nuclear material) and cellularity. Mitotic figures and necrosis are additional features useful in grading.

Two cases of normal cartilage were obtained from condylar cartilage of a mandible recommended for hemimandibulectomy due to evidence of squamous cell carcinoma lesion.

### I- Clinical study

The clinical data were collected retrospectively through the computerized retrospective database from the tumor registry, regarding age, sex, site and recurrence.

### II- Histological study

Sections of 4 μm thickness were cut, deparaffinized, rehydrated and stained with (a) hematoxylin and eosin for re-evaluation and confirmation of histopathological malignancy grading diagnosis and (b) for the immunohistochemical evaluation of MMP-13 expression.

### III- Immunohistochemical study

Paraffin sections were employed for immunostaining for collagenase-3 (MMP-13) (mouse monocolonal antibody) clone (lab vision corporation VIII A2), by the standard avidin-biotin peroxidase complex technique (ABC) (Vectastain ABC kit; Vector Laboratories, Burlingame, CA, USA), according to Hsu et al., [46]. Sections were rehydrated by passing through xylene and a series of graded alcohols (90% to 70%), then to water. The rehydrated slides were treated sequentially with 0.3% hydrogen peroxide (H2O2) in methanol for 30 minutes to block endogenous peroxidase activity. The slides were placed in 10 mM citric acid buffer at pH 6.0 and underwent antigen retrieval for 10 min at 750 w and 95°c in a microwave oven followed by cooling at room temperature for 20 minutes. According to the manufacturer, the primary antibody MMP-13 (cat. ≠ 825- R7) is ready to use (7.0 ml), applied on the sections, and stored overnight at 4°c. Then, the slides were incubated with the second biotinylated antibody and the avidin-biotin complex reagent (Vector Laboratories, Burlingame, CA, USA). After 30 min at room temperature, the reaction was developed with using diaminobenzidine (DAB) as chromogen. The sections were then counterstained with Mayer's hematoxylin before mounting.

To guard against false negative and false positive results, negative and positive control sections were used in each batch.

Negative control sections were performed by substituting non-immune serum for MMP-13. Positive control sections of human breast carcinoma were also included.

### Staining assessment

Quantitation of immunoreactivity was performed using an Olympus light microscope interfaced via a Sony camera to an image analysis system (Qwin Pro, Leica, Wetzlar, Germany). The percentages of collagenase-3. immunopositive cells were obtained from 20 random fields per case/section using a 10× objective lens.

Semiquantitative estimation of collagenase-3 immunostaining intensities was made in immunopositive cells by arbitrarily assigning as follows: negative (-) = 100% of cells negative, mild (+) = (0% – < 25%) of cells positive, moderate (++) = (25% – >50%) of cells positive and intense (+++) = (75% – 100%) of the cells positive.

The staining intensity in MMP-13 positive cases was verified as follows:

• Strong: Densely stained reaction visible at low magnification (objective 4×).

• Weak: faintly cytoplasmic reaction only visible using a higher magnification.

### Statistical analysis

Data were analyzed for significance of difference by chi-square analysis of 2 × 2 contingency tables by the Kwikstat computer program (Texa soft software) [47]. P-value is considered significant when P ≤ 0.05.

## Results

### Clinical findings

In this study, eleven cases of chondrosarcoma of the jaws were examined. The age of our patients ranged between 14–68 years with a mean of 39.7 years. Regarding the sex, seven patients were males and four females. The most frequent site of involvement was the mandible (eight cases) followed in frequency by the maxilla, in the anterior region (three cases). Three of whom had a recurrence.

### Histopathological evaluation

**Figure 1:**
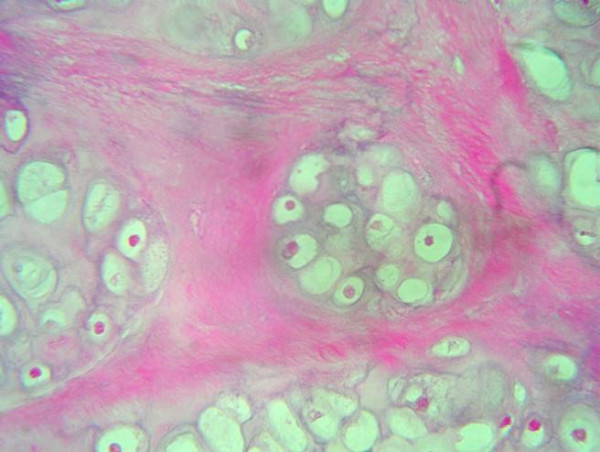
Lobules of malignant chondrocytes in chondroid matrix (H & E stain).

**Figure 2:**
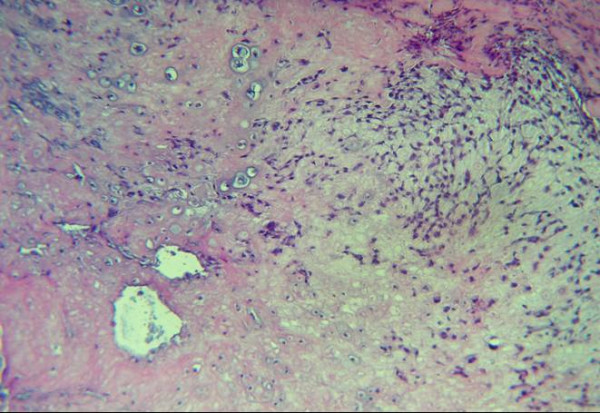
Variation in the size of chondrocytes nuclei in the lacunae (H &E stain).

**Figure 3:**
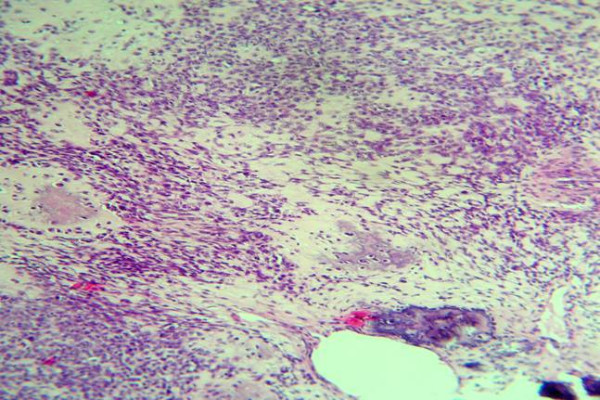
Foci of malignant cartilage with anaplastic hyperchromatic pleomorphic cells dispersed in myxoid stroma (H &E stain).

Regarding the grading of chondrosarcoma, there were four cases grade I (Fig. 1), five cases grade II (Fig. 2), and two cases grade III (Fig. 3).

On microscopic analysis, lower grade chondrosarcomas composed of lobules of chondrocytes in a chondriod background, separated by fibrous septa (Fig. 1). By contrast, higher-grade lesions tend to harbor regions of densely packed hyperchromatic malignant looking cells. Myxoid changes or chondroid matrix liquefaction is a common feature of chondrosarcomas particularly in Grade II and Grade III lesions. Grade II (or intermediate grade) is more cellular with a greater degree of nuclear atypia, hyperchromasia and nuclear size (Fig. 2). Grade III (or high grade) tumors have significant areas of marked pleomorphism, large cells with more hyperchromatic nuclei than grade II (Fig. 3). Occasional giant cells, abundant necrosis and mitoses are frequently detected.

Mitotic counts were between 2 and 35 per 10 high power field (mean = 8.5). Necrosis was present in 7 of cases: less than 50% in 4 tumors and more than 50% in 3 tumors.

### Immunohistochemical findings

#### Pattern of MMP-13 immunostaining

**Figure 4:**
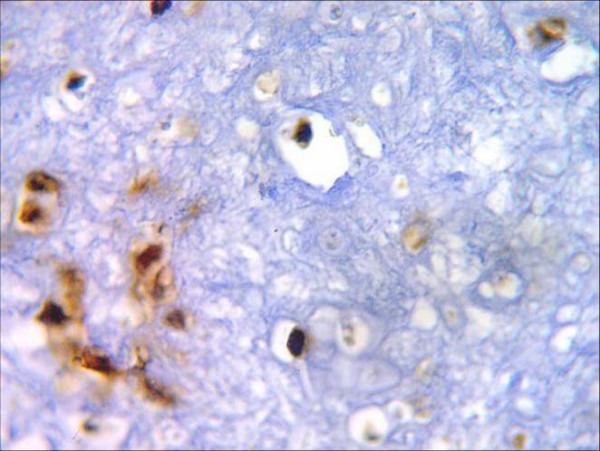
Moderate positive nuclear and cytoplasmic MMP-13 immunoreactivity in malignant chondrocytes (ABC DAB).

The positive immunohistochemical reactivity to MMP-13 appeared as brown cytoplasmic and nuclear staining reaction was also identified in tumor cells (Fig. 4). There was no positive staining for MMP-13 observed in normal cartilage.

Table 1 summarizes the intensity and percentage of immunoreactive cells for the MMP-13 detected in the present series. The overall immunoreactivity among the currently employed chondrosarcomas was identified in eight of eleven cases (72.8%). Only three cases failed to reveal staining for MMP-13. Malignant chondrocytes expression of MMP-13 was limited almost entirely to high-grade lesions (grade II and grade III).

**Table 1:** Immunohistochemical staining reaction for MMP-13 in normal cartilage and chondrocarcomas.

Samples	No of cases	Intensity of staining				Percentage of positive cells (%)
			
		Negative -	Mild +	Moderate ++	Intense +++	
Normal cartilage	2	2 (100%)	-	-	-	-
GI chondrosarcoma	4	3 (75%)	1 (25%)	-	-	0 %–4.2 %
GII chondrosarcoma	5	-	-	2 (40%)	3 (60%)	15.4%–30.8%
GIII chondrosarcoma	2	-	-	-	2 (100%)	37.6%–40.2%

(-) Negative : 100% of cells negative.

(+) Mild : 0% – < 25% of cells positive.

(++) Moderate : 25% – >50% of cells positive.

(+++) Intense : 75% – 100% of cells positive

**Figure 5:**
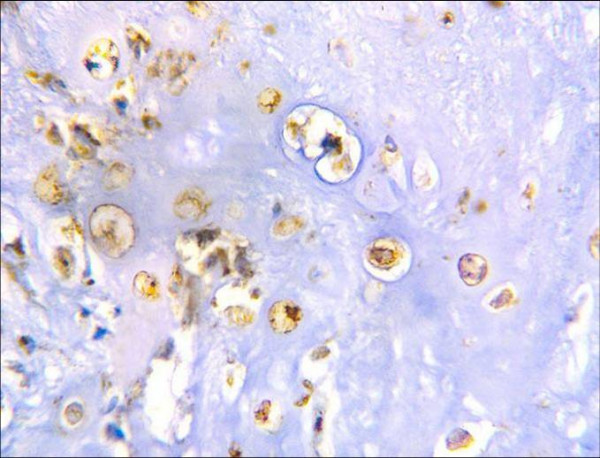
Grade I chondrosarcoma shows mild positive cytoplasmic and nuclear reaction to MMP-13 (ABC DAB).

In grade I chondrosarcomas, one of four cases revealed mild staining for MMP-13 (Fig. 5), while the other three cases showed negative reaction.

**Figure 6:**
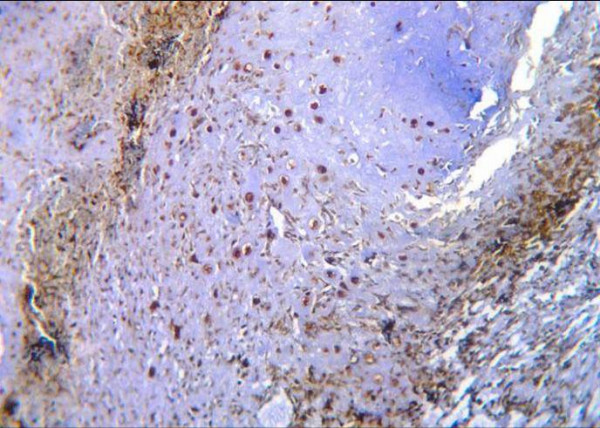
Diffuse strong positive cytoplasmic & nuclear reaction to MMP-13 in neoplastic chondrocytes (ABC DAB).

**Figure 7:**
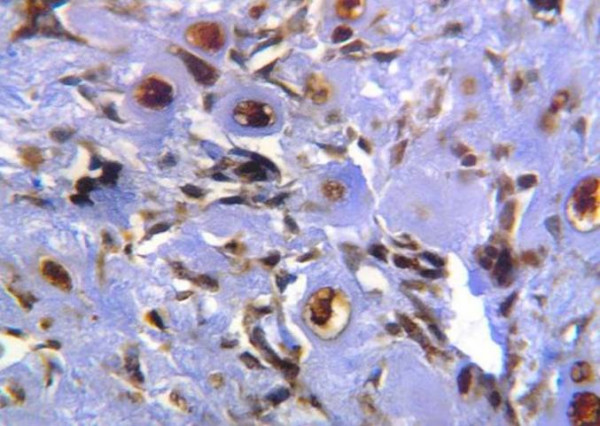
Intense MMP-13 reaction in proliferating neoplastic chondrocytes located near blood vessels (ABC DAB).

In grade II chondrosarcomas, three of five cases showed strong immunoreaction for MMP-13 (Fig. 6&7). While the last two cases revealed moderate immunoreactivity.

**Figure 8:**
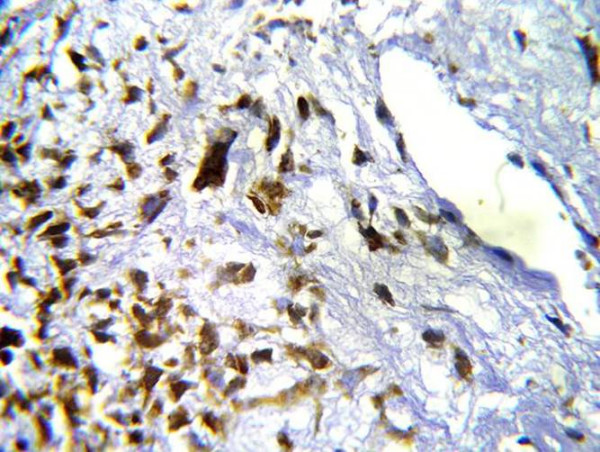
Strong positive cytoplasmic and nuclear reaction for MMP-13 in highly anaplastic undifferentiated cells (ABC DAB).

In grade III chondrosarcomas, only two cases were examined and revealed strong MMP-13 immunoreactivity (Fig. 8).

**Figure 9:**
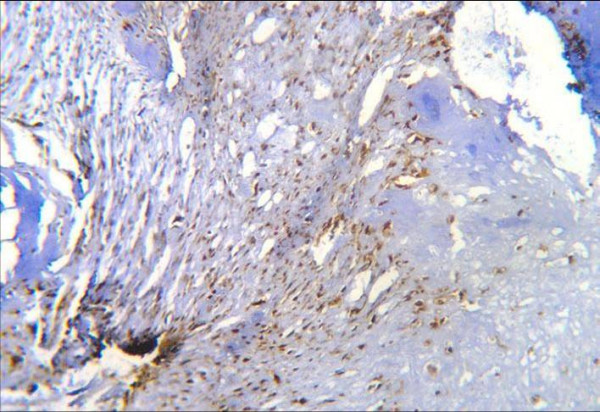
Moderate positive reaction for MMP-13 in malignant chondrocytes located near blood vessels (ABC DAB).

The immunoreactive pattern of MMP-13 on chondrosarcoma sections showed variable in intensity and percentage of positive cells in different fields in the same case (Fig. 4). Moderate to strong MMP-13 immunoreactivity was observed in the chondrocytes that were located near blood vessels (Fig. 7 &9).

### Relation between MMP-13 expression and clinico-pathological parameters of chondrosarcomas (Table 2)

**Table 2:** Correlation between MMP-13 expression and clinicopathological parameters of chondrosarcoma patients.

**Parameter**	**No. of patients**	**MMP-13 immunostaining**		**P-value**
			
		**MMP-13 (-ve) (n = 3) %**	**MMP-13 (+ve) (n = 8) %**	
Age				
>60	6	2 (33.3%)	4(66.7%)	
≤ 60	5	1 (20%)	4 (80%)	NS
Sex				
Male	7	2 (28.6%)	5(71.4%)	
Female	4	1 (25%)	3(75%)	NS
Site				
Mandible	8	2 (25%)	6(75%)	
Maxilla	3	1 (33.3%)	2(66.7%)	NS
Mitosis				
0	3	3 (100%)	0(0%)	
1	4	0(0%)	4(100%)	
2	2	0(0%)	2(100%)	*S
3	2	0 (0%)	2 (100%)	
Necrosis				
o	5	3 (60%)	2 (40%)	
1	4	0 (0%)	4 (100%)	*S
2	2	0 (0%)	2 (100%)	
Grades				
G-I	4	3 (75%)	1 (25%)	
G-II	5	0 (0%)	5 (100%)	*S
G-III	2	0 (0%)	2 (100%)	
Recurrence				
+	3	0(0%)	3 (100%)	
-	8	3 (37.5%)	5 (62.5%)	NS

P ≤ 0.05 means significant difference

*S = Signficant

NS = Not significant

Necrosis Non = "0", <50% = "1", ≥ 50% = "2".

Mitosis 0–9/10HPF = "1", 10–19/10HPF = "2", ≥ 20/10HPF = "3"

The correlation between MMP-13 expression and clinicopathological parameters of the jaws chondrosarcoma cases is summarized Table 2. There was no significant difference between MMP-13 positive and negative groups regarding to age and sex of the patients as well as tumor site. In contrast, there was significant difference (P < 0.05) between the two groups concerning the mitotic counts and necrosis. Comparing the intensity of MMP-13 reaction between low and high grade chondrosarcomas revealed that the difference was significant (P < 0.05). Regarding the recurrence, the highest expression of MMP-13 was observed in all the examined specimens. However, there was no significant correlation between MMP-13 expression in primary lesions and their local recurrence.

## Discussion

Human collagenase-3 (MMP-13), a new member of the MMP family, is expressed by breast tumors [30]. It is a powerful collagenolytic and gelatinolytic enzyme that preferentially cleaves type II collagen and it can therefore be implied that this enzyme may play a considerable role in connective tissue turnover [32]. MMP-13 plays an important role in tumor progression in numerous human malignancies [48], including colorectal cancer [49], breast cancer [30], squamous cell carcinomas [36,37], and melanoma [50].

Absence of MMP-13 expression in normal cartilage [41-51] may be explained by the destruction of MMP-13 by other peptidases related to digestion or possibly due to the very low rate of its synthesis in this tissue [52]. Also, it might be due to the prolonged duration of the slow process of ossification that is preceded by cartilage matrix degradation by this collagenase [41,42]. Normally, MMP-13 expression by chondrocytes appears to be limited to cartilage development. Chondrocytes produce MMP-13 to remove type II collagen at the growth plate during bone formation [41,42]. Whereas neoplastic chondrocytes were the most immunoreactive cells in the tumor sections examined. The involvement of chondrocytes in MMP-13 production in chondrosarcoma of jaws is related to their roles in matrix turnover [32-52]. Chondrocytes maintain the integrity of the cartilage in normal joints, but in the pathological conditions, chondrocytes are influenced to change their properties by the action of inflammatory cytokines to begin secreting matrix-degrading enzymes and to eventually cause tumor. Therefore, the relative role of collagenase-3 in chondrosarcoma becomes important [53].

In the current study, it is of interest that, despite the fact that most of analyzed chondrosarcoma cases were positive for MMP-13 expression, there were clear variations in the percentage of positive cells and the intensity of staining. The highest collagenase-3 (MMP-13) positive immunoreactivity was found among grade III chondrosarcoma cases. While, the weakest reaction for MMP-13 was noted in most cases of grade I chondrosarcoma. In accordance with Uria et al [54], these variation in MMP-13 staining intensity may be dependant on the biological behavior and histopathological features (degree of cellularity and pleomorphism) in this tumor.

Staining for MMP-13 is usually cytoplasmic but nuclear staining was also seen in some cases. This finding was in accordance with the results of other reports [49], its significance is undetermined, but its presence warrants documentation. This observation may be attributed to MMP-13 Ab used here is over expressed in its early stages in the nucleus or it may possibly due to alteration in the processing or stability of MMP-13. Strong expression of cytoplasmic and nuclear MMP-13 was observed with high grade tumors. Thus, nuclear MMP-13 staining may help to identify a category with worse prognosis in this tumor.

Strong immunoreactivity of MMP-13 in chondrocytes located near blood vessels may be due to increased response of malignant chondrocytes to the inductive effect of the stimulatory factors produced by endothelial cells such as different cytokines (IL-1β, TGF-β or TNF-α) [18-56]. These factors were previously found to play important roles in the induction of collagenase-3 in fibroblasts, keratinocytes, or primary chondrocytes [39-53].

MMP-13 is over-expressed in breast carcinomas but not in normal mammary gland nor in benign mammary lesions [30-58]. Additional reports revealed similar results among squamous cell carcinomas [18-56] and malignant melanoma [50]. This observation suggests that up-regulation of MMP-13 may be somewhat linked to the malignant transformation and contribute to the progression of these tumors [54]. MMP-13 could play a critical role in the uncontrolled lytic processes occurring during malignant progression. This possibility is consistent with biochemical properties of this enzyme which has been characterized as a potent proteinase with a wide substrate specificity including a preferential degrading activity on type II collagen [31-44] which is the most abundant type in articular cartilage [60,61].

The increase in MMP-13 expression may be necessary for the activation of other MMPs that have previously been identified in chondrosarcoma such as MMP-2 and MMP-9 [62] it is also activated by MMP-2, MMP-3, and MTI-MMP [32]. Furthermore, it is logical that tumors have far higher amounts of MMP-13 activity because this molecule catalyses the breakdown of ECM necessary for invasion. Similarly, normal chondrocytes have no requirement for MMP-13 mediated ECM breakdown, or increased activation of other MMPs. These findings again strengthen the link between MMP-13 expression and tumor progression.

The mechanisms involved in MMP-13 activation are complex. Previous investigations reported that the production of a growth factor called basic fibroblast growth factor (bFGF) by the malignant chondrocytes that stimulates the same cell to produce collagenase-3 (MMP-13) [23-64]. This factor might be responsible for the currently observed increase expression of MMP-13 among the high-grade cases of chondrosarcoma of jaws. The precise molecular mechanisms responsible for collagenase-3 up-regulation in chondrosarcoma cells in response to bFGF have not yet been elucidated, although it is likely that members of the FGF receptor family may be involved [62-65]. The availability of a phenotypically stable chondrosarcoma cell line with ability to produce collagenase-3 after bFGF treatment will be very helpful for further evaluation. The precise mechanisms mediating the bFGF elicited induction of this MMP gene in human chondrosarcoma [54]. These variations need further investigations to reveal possible implication to the clinical outcome.

Fong et al., [40] found that there was no significant association between MMP-13 expression and patient's age, tumor site and local recurrence in chondrosarcomas. This result was in accordance with our results.

A finding of particular interest in this study was the occurrence of positive MMP-13 in most of the mitotically active cells. This finding may reflect the high expression of MMP-13 in the cases with high score of mitosis. Accordingly, it can be claimed that the presence of MMP-13 might be associated with proliferative activity and tumor aggressiveness [50].

A significant association between MMP-13 expression and patient's poor prognosis was observed in this study. This finding was in agreement with previous reports on breast carcinoma [35], oral squamous cell carcinoma [37] and malignant melanoma [50]. On the contrary, others [14] have found no correlation between MMP-13 over-expression and prognosis in chondrosarcoma. This confliction can be attributed to variables in ethnic and environmental factors influencing the tumor growth in different populations. However, the number of the cases that examined in the present work is small to consider this a true confliction.

The ability to predict the biologic behavior of cartilaginous neoplasms has been difficult and many studies have sought to identify a marker for recurrence [66,67]. The expression of MMP-13 was present in all the examined specimens. However, it could not be correlated with disease recurrence. This result was in agreement with Scully et al., [68] who found that the expression of MMP-13 has no prognostic significance for recurrence in chondrosarcoma. It has been reported that as a tumor dedifferentiates and becomes more aggressive biologically, there is a phenotypic drift that results in increased amounts of type I collagen being synthesized [69,70]. It is possible that specificity for cleavage of type I collagen may be more important than that of type II collagen to permit cell egression and this is what leads to the observed results.

The understanding of cancer pathogenesis is rapidly growing and evidence indicates that several molecular genetic markers are involved in the initiation and/or progression of the disease. The interactions of the molecular genetic changes are complex; however, some may be important targets for the development of specific inhibitors to serve as potential chemotherapeutic agents. An extracellular matrix and basement membrane turnover is regulated by a delicate balance between the molecular factors that participate in the production, activation, and inhibition of MMPs and the production of natural tissue inhibitors of matrix metalloproteinases (TIMPs) [71]. Because of their central role in tumor invasion and metastasis, MMPs are potential targets for therapeutic intervention. Several investigations have demonstrated the effectiveness of anti-MMP therapy [72]. A synthetic small molecule has been shown to inhibit the endotoxin and cytokine-induced synthesis of MMP [73]. The inhibition of MMPs such as MMP-13 has produced encouraging results by decreasing the number, size and speed of development of metastatic nodules in animals [74]. In the present study, the MMP-13 expression was detected in 72.8 % of the samples. The fact that MMP-13 activation is likely to occur in chondroasrcoma of the jaws may make it particularly suitable for treatment with the emerging class of drugs to block MMP-13 secretion and matrix invasion. Using MMP-13 inhibition as one arm of a multitargeted approach will have benefits for the treatment of chondroasrcoma of the jaws, when a larger series of studies confirms MMP-13 representation in these tumors. In addition, considering the many factors involved such as tissue inhibitors of metalloproteinases (TIMPs) or various stimulating factors for MMPs, the relationship between chondroasrcoma of the jaws and TIMPs and other subtypes of MMPs in worthy of further studies.

## Conclusion

**1- **On the basis of positive expression of MMP-13 in the majority of chondrosarcoma of the jaws and absence of detectable expression in normal cartilage, a possible role for this metalloproteinase in the tumoral process is proposed.

**2- **The present study provides evidence of the prognostic significance of MMP-13 in chondrosarcoma of the jaws where the association between this marker and mitotic counts, necrosis and high grade tumors suggests the possible role of MMP-13 in determining the highly aggressive tumors.

**3- **It may become a therapeutic target for jaws chondrosarcoma's patients with MMP-13 positive tumor.

These results are preliminary because of the small sample size and should be verified in a larger number of cases. Further exploration of these findings and correlation with clinical follow-up are needed to corroborate these associations. Similarly, it will be of interest to examine whether the small percentage of benign lesions producing MMP-13 could be indicative of an increased risk of malignant transformation in these patients.
